# The relationship between weight-adjusted waist index and peripheral artery disease

**DOI:** 10.3389/fnut.2025.1504896

**Published:** 2025-02-12

**Authors:** Zhe Wu, Yang Liu, Bin Wang

**Affiliations:** ^1^The First Clinical College, Shandong University of Traditional Chinese Medicine, Jinan, China; ^2^Department of General Surgery, Vascular Surgery, Qilu Hospital of Shandong University, Jinan, China; ^3^Department of Vascular Surgery, The Second Affiliated Hospital of Shandong University of Traditional Chinese Medicine, Jinan, China

**Keywords:** weight-adjusted waist index, peripheral artery disease, NHANES, obesity, cross-sectional study

## Abstract

**Background:**

Obesity is a significant risk factor for peripheral arterial disease (PAD). The weight-adjusted waist index (WWI) is a novel obesity metric that better reflects abdominal obesity than traditional body mass index (BMI). However, research on the relationship between WWI and PAD remains scarce.

**Methods:**

Relevant data from the NHANES 1999–2004 were selected. Multiple logistic regression and restricted cubic spline (RCS) analyses were used to assess the relationship between WWI and the risk of PAD. Additionally, the area under the curve (AUC) of the receiver operating characteristic (ROC) was used to evaluate the predictive ability of WWI for PAD.

**Results:**

A total of 5,686 participants were included in the study, of whom 476 had PAD and 5,210 did not. The results of multiple logistic regression showed that WWI was significantly positively associated with the risk of PAD after full adjustment for covariates (OR: 1.407, 95% CI: 1.100–1.799). Additionally, compared to the first quartile of WWI, the risk of PAD significantly increased in the second quartile (OR: 2.042, 95% CI: 1.333–3.129), third quartile (OR: 2.134, 95% CI: 1.354–3.364), and fourth quartile (OR: 2.491, 95% CI: 1.435–4.325). The ROC results showed that the AUC value for WWI was 0.697, while the AUC value for BMI was 0.520. Compared to BMI, WWI has a better predictive value for the risk of PAD.

**Conclusion:**

There is a significant positive correlation between WWI and the risk of PAD. For individuals with high WWI, efforts should be made to reduce WWI to prevent the onset of PAD.

## Introduction

Peripheral artery disease (PAD) is a form of atherosclerotic disease characterized by the narrowing or occlusion of arteries in the lower extremities, leading to ischemic symptoms such as intermittent claudication or rest pain (1–6). With the global population aging and the incidence of metabolic diseases increasing, the prevalence of PAD is also on the rise. It is estimated that more than 200 million people worldwide suffer from PAD, with a prevalence of up to 3.62% among individuals aged 40 and above (7, 8). PAD is closely associated with an increased risk of amputations, as well as cardiovascular events such as myocardial infarction and stroke, making it a major global public health issue (9, 10). Despite its wide-ranging and profound impact on health, PAD remains a condition that is underdiagnosed and undertreated (11). In the early stages, PAD often presents with few or no obvious symptoms or may only manifest as intermittent claudication, which can be easily confused with conditions such as lumbar disk herniation (12). Moreover, current pharmacological treatments for PAD mainly target intermittent claudication (12, 13). As the condition progresses, patients may develop foot ulcers, which can even worsen to gangrene. At this time, conventional treatment methods are often unable to meet the patient’s treatment needs. Exploring risk factors for PAD, especially modifiable ones, is crucial for reducing the disease burden and improving patient outcomes.

In the United States, the prevalence of obesity is constantly increasing. The prevalence of obesity among Americans aged 40 years and over is more than 40% (14). Multiple studies have shown a significant correlation between obesity and the incidence and mortality of peripheral arterial disease and various cardiovascular diseases (15–18). Body mass index (BMI) is frequently used as a measure of obesity in studies of cardiovascular and metabolic diseases (19). BMI also has its limitations. It can only reflect general obesity and cannot distinguish specific fat distribution. Compared to BMI, the weight-adjusted waist index (WWI) better reflects abdominal obesity, which is an important risk factor for metabolic disorders and cardiovascular disease (20–22). In recent years, studies have begun to examine the relationship between WWI and cardiovascular disease, finding a positive association with an increased risk of cardiovascular diseases such as coronary heart disease and hypertension (23–26). Abdominal obesity can promote atherosclerosis through mechanisms such as inflammation, metabolic disturbances, and vascular dysfunction, thereby increasing the risk of PAD (27–29). However, no studies have yet examined the relationship between WWI and PAD.

This study aims to explore the relationship between WWI and PAD by analyzing relevant data from NHANES 1999 to 2004, providing new perspectives and evidence for PAD risk prediction and intervention.

## Methods

### Data source

NHANES is a nationally representative survey conducted by the National Center for Health Statistics (NCHS) in the United States. The data related to our research are taken from NHANES 1999 to 2004.

### Inclusion criteria

The inclusion criteria include: (1) participants who voluntarily participate in relevant NHANES research and sign a written informed consent form, (2) participants who are 40 years old or above, and (3) participants who have undergone examinations related to lower limb diseases.

### Exclusion criteria

The exclusion criteria include: (1) participants with missing WWI data, (2) participants with missing available ankle-brachial index (ABI) data, (3) participants with ABI > 1.4, and (4) participants with missing relevant covariates.

### Peripheral artery disease

Systolic blood pressure was measured in the brachial artery of the right arm, or the left arm if the right arm could not be measured or if the results were affected. Ankle systolic pressure was measured in the posterior tibial artery on both sides. The ankle systolic pressure for each side was divided by the arm systolic pressure to obtain the ABI for both sides. PAD was diagnosed if the ABI in at least one leg was less than 0.9.

### WWI

The WWI was calculated by dividing waist circumference in centimeters by the square root of body weight in kilograms (30). All measurements were conducted by professionally trained medical personnel to ensure the reliability of the results.

### Covariates

Covariates included age, gender, ethnicity, poverty income ratio (PIR), education, marital status, albumin, total cholesterol, aspartate aminotransferase (AST), alanine aminotransferase (ALT), smoking history, and whether participants had hypertension, diabetes, or chronic kidney disease (CKD). Hypertension was defined as an average systolic blood pressure ≥ 140 mmHg and an average diastolic blood pressure ≥ 90 mmHg, or as a diagnosis by a physician or the use of antihypertensive medication. Diabetes was defined as fasting blood glucose ≥7 mmol/L, random blood glucose ≥11.1 mmol/L, 2-h OGTT blood glucose ≥11.1 mmol/L, glycated hemoglobin ≥6.5%, or a physician diagnosis or use of hypoglycemic medication. CKD was defined as an estimated glomerular filtration rate < 60 mL/min/1.73 m^2^.

## Statistical analysis

Statistical analysis was conducted using R Studio (version 4.2.1). All analyses were weighted according to population data. Continuous variables are represented as mean (standard error), and categorical variables are represented as numbers (weighted percentage). Multiple logistic regression was used to explore the relationship between WWI and PAD, and restricted cubic splines (RCS) were used to assess the dose–response relationship. WWI levels were categorized into quartiles: Q1 ≤ 10.614, 10.614 < Q2 ≤ 11.105, 11.105 < Q3 ≤ 11.637, and Q4 > 11.637. Subgroup analyses were conducted based on age, gender, ethnicity, PIR, education, marital status, smoking history, and the presence of hypertension, diabetes, and CKD. The likelihood ratio test was used to detect interactions. In addition, the receiver operating characteristic (ROC) curve was used to determine the predictive ability of WWI for the risk of PAD.

## Results

### Baseline characteristics of the study population

**Figure 1:**
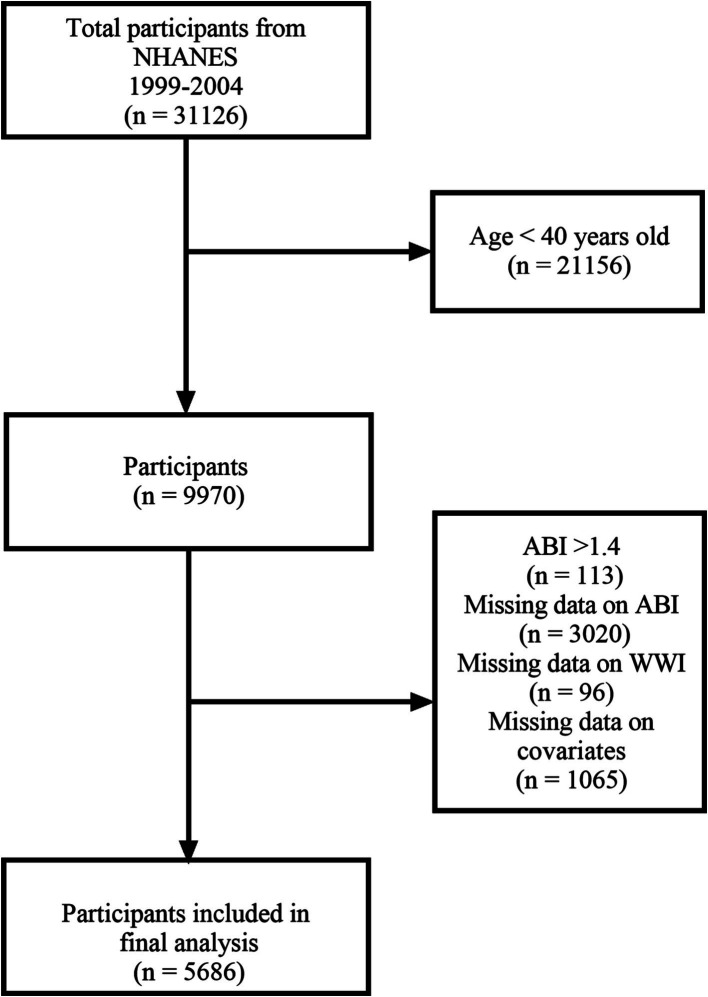
Research flowchart.

From 1999 to 2004, 31,126 participants took part in the NHANES, with 9,970 participants aged 40 and above undergoing lower extremity disease tests. We excluded participants with an ABI >1.4 (*n* = 113) and those with missing ABI data (*n* = 3,020). Additionally, we excluded participants with missing WWI data (*n* = 96) and missing covariate data (*n* = 1,065). Ultimately, 5,686 participants were included in the study (Figure 1).

Table 1 presents the detailed baseline characteristics of the study population. Participants were divided into two groups based on the presence of PAD, with 476 participants diagnosed with PAD and 5,210 without PAD. Significant differences were observed between the two groups in terms of age, ethnicity, PIR, education, marital status, WWI, albumin, ALT, AST, smoking history, and the prevalence of hypertension, diabetes, and CKD.

**Table 1:** Population characteristics stratified by PAD.

Variable	Non-PAD (*n* = 5,210)	PAD (*n* = 476)	*p*-value
Age (years)	54.918 (0.232)	67.828 (0.767)	< 0.001
Sex			0.186
Male	2,692 (49.152)	240 (44.518)	
Female	2,518 (50.848)	236 (55.482)	
Ethnicity			< 0.001
White	2,902 (78.814)	276 (79.118)	
Black	838 (7.907)	105 (13.575)	
Mexican American	1,105 (4.640)	75 (3.324)	
Other ethnicities	365 (8.638)	20 (3.984)	
PIR			< 0.001
< 1.3	1,263 (15.954)	160 (27.920)	
≥ 1.3, < 3	1,574 (24.944)	196 (38.388)	
≥ 3	2,373 (59.102)	120 (33.692)	
Education			< 0.001
< High school diploma	849 (6.521)	121 (14.024)	
High school diploma	1981 (37.243)	209 (47.073)	
> High school diploma	2,380 (56.236)	146 (38.903)	
Marital status			< 0.001
Married	3,355 (68.343)	247 (55.554)	
Not married	1855 (31.657)	229 (44.446)	
WWI	10.956 (0.018)	11.490 (0.056)	< 0.001
Albumin (g/dL)	4.301 (0.008)	4.175 (0.023)	< 0.001
ALT (u/L)	26.703 (0.329)	21.422 (0.708)	< 0.001
AST (u/L)	25.792 (0.309)	23.894 (0.638)	0.017
Total cholesterol (mg/dL)	211.035 (0.956)	206.924 (2.635)	0.112
Smoking history			< 0.001
No	2,462 (47.106)	155 (31.686)	
Yes	2,748 (52.894)	321 (68.314)	
Diabetes			< 0.001
No	4,405 (88.883)	329 (72.910)	
Yes	805 (11.117)	147 (27.090)	
Hypertension			< 0.001
No	2,546 (55.247)	109 (26.020)	
Yes	2,664 (44.753)	367 (73.980)	
CKD			< 0.001
No	4,669 (92.262)	306 (66.567)	
Yes	541 (7.738)	170 (33.433)	

### Multivariate logistic regression

To further verify the relationship between WWI and PAD, we conducted a multivariate logistic regression analysis. The results showed that WWI was significantly positively associated with the risk of PAD in unadjusted models (OR: 2.524, 95% CI: 2.040–3.123), models adjusted for age, gender, and ethnicity (OR: 1.683, 95% CI: 1.307–2.168), and fully adjusted models (OR: 1.407, 95% CI: 1.100–1.799). Additionally, after full adjustment for covariates, the risk of PAD was found to be significantly higher in the second quartile (OR: 2.042, 95% CI: 1.333–3.129), third quartile (OR: 2.134, 95% CI: 1.354–3.364), and fourth quartile (OR: 2.491, 95% CI: 1.435–4.325) (Table 2).

**Table 2:** Relationship between WWI and PAD.

Result	Model 1		Model 2		Model 3	
	OR (95%CI)	*P*-value	OR (95%CI)	*P*-value	OR (95%CI)	*P*-value
WWI	2.524 (2.040,3.123)	<0.001	1.683 (1.307,2.168)	<0.001	1.407 (1.100,1.799)	0.008
Q1	ref	ref	ref	ref	ref	ref
Q2	2.504 (1.698, 3.695)	<0.001	2.002 (1.325,3.026)	0.002	2.042 (1.333,3.129)	0.002
Q3	3.880 (2.534, 5.941)	<0.001	2.265 (1.444,3.555)	<0.001	2.134 (1.354,3.364)	0.002
Q4	7.615 (4.831,12.003)	<0.001	3.100 (1.812,5.304)	<0.001	2.491 (1.435,4.325)	0.002

Adjusted variables: Model 1: unadjusted. Model 2: age, sex, ethnicity, PIR, education, marital status. Model 3: age, sex, ethnicity, PIR, education, marital status, smoking history, albumin, total cholesterol, ALT, AST, diabetes, hypertension, CKD.

### Restricted cubic splines

**Figure 2:**
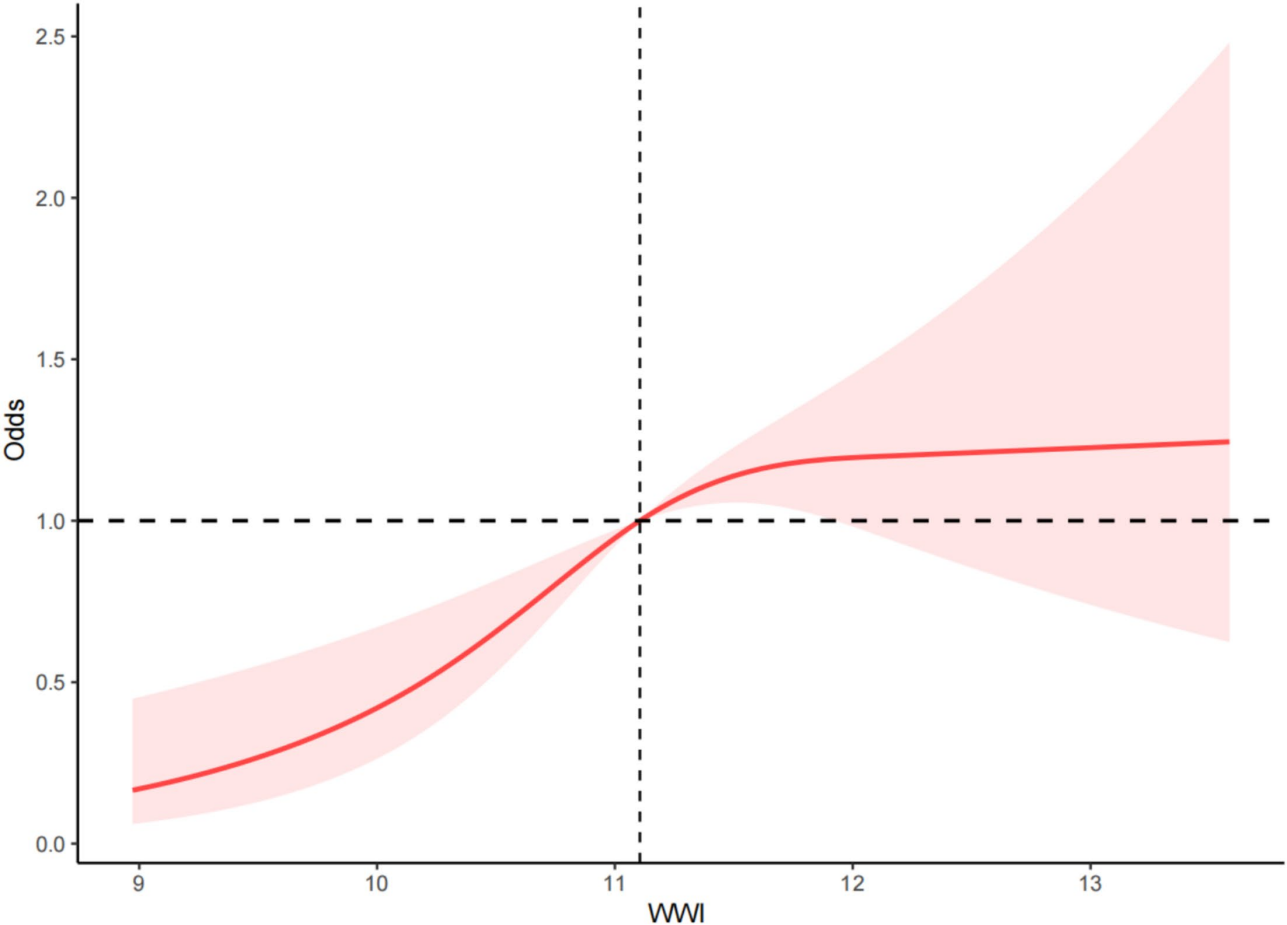
Dose–response relationship between WWI and PAD.

RCS was used to examine the dose–response relationship between WWI and PAD. The RCS results indicated a non-linear positive correlation between WWI and the risk of PAD (*p* < 0.001, P for non-linearity = 0.019) (Figure 2).

### ROC curves of WWI and BMI in relation to PAD

**Figure 3:**
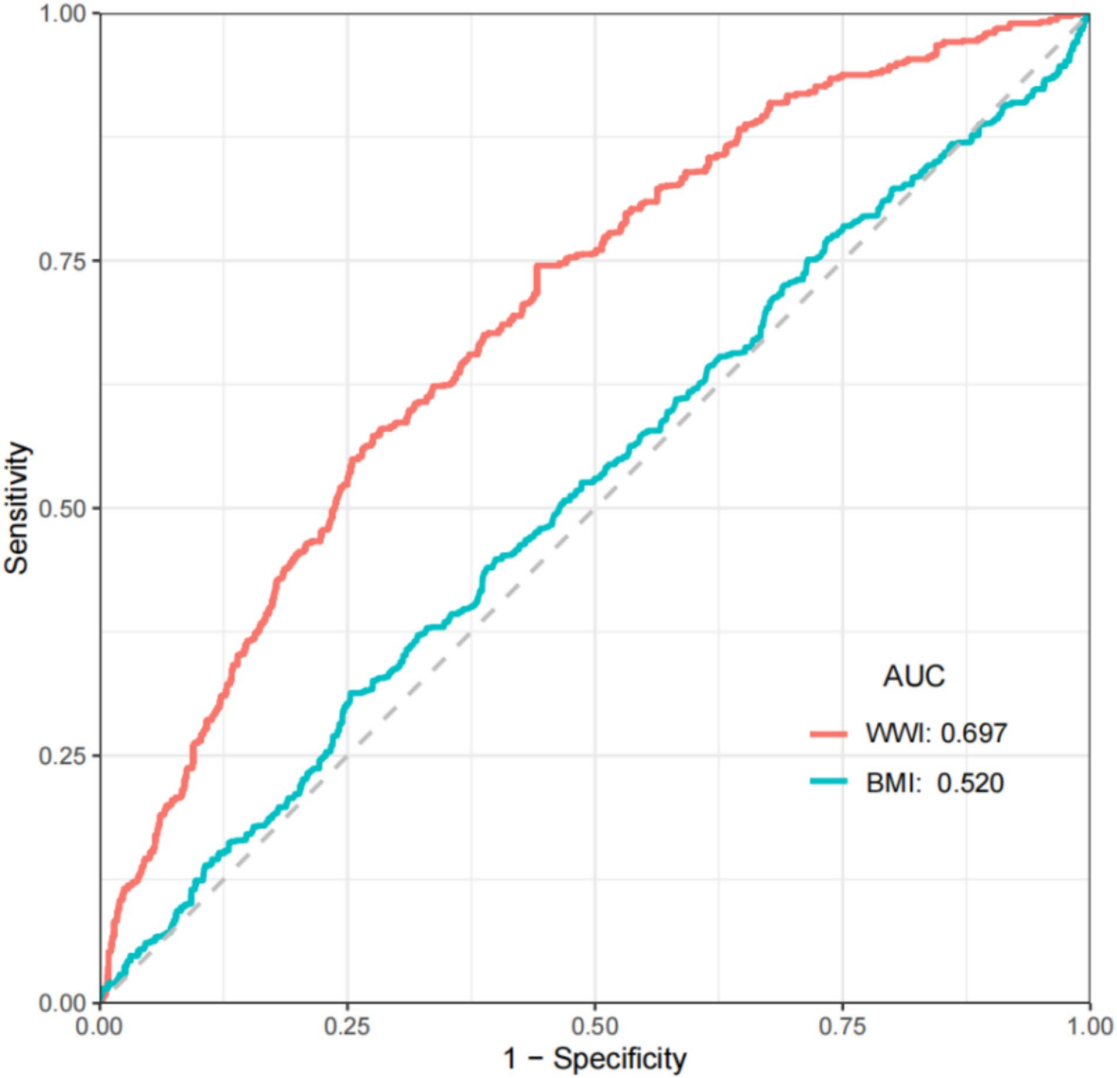
ROC curves analysis of WWI and BMI in relation to PAD.

We perform ROC analysis based on weighted data. The results showed that the AUC value of BMI was only 0.520, while the AUC value of WWI was 0.697. (Figure 3) Compared to BMI, WWI exhibited higher diagnostic efficiency and specificity in predicting the risk of PAD.

### Sensitivity analysis

**Figure 4:**
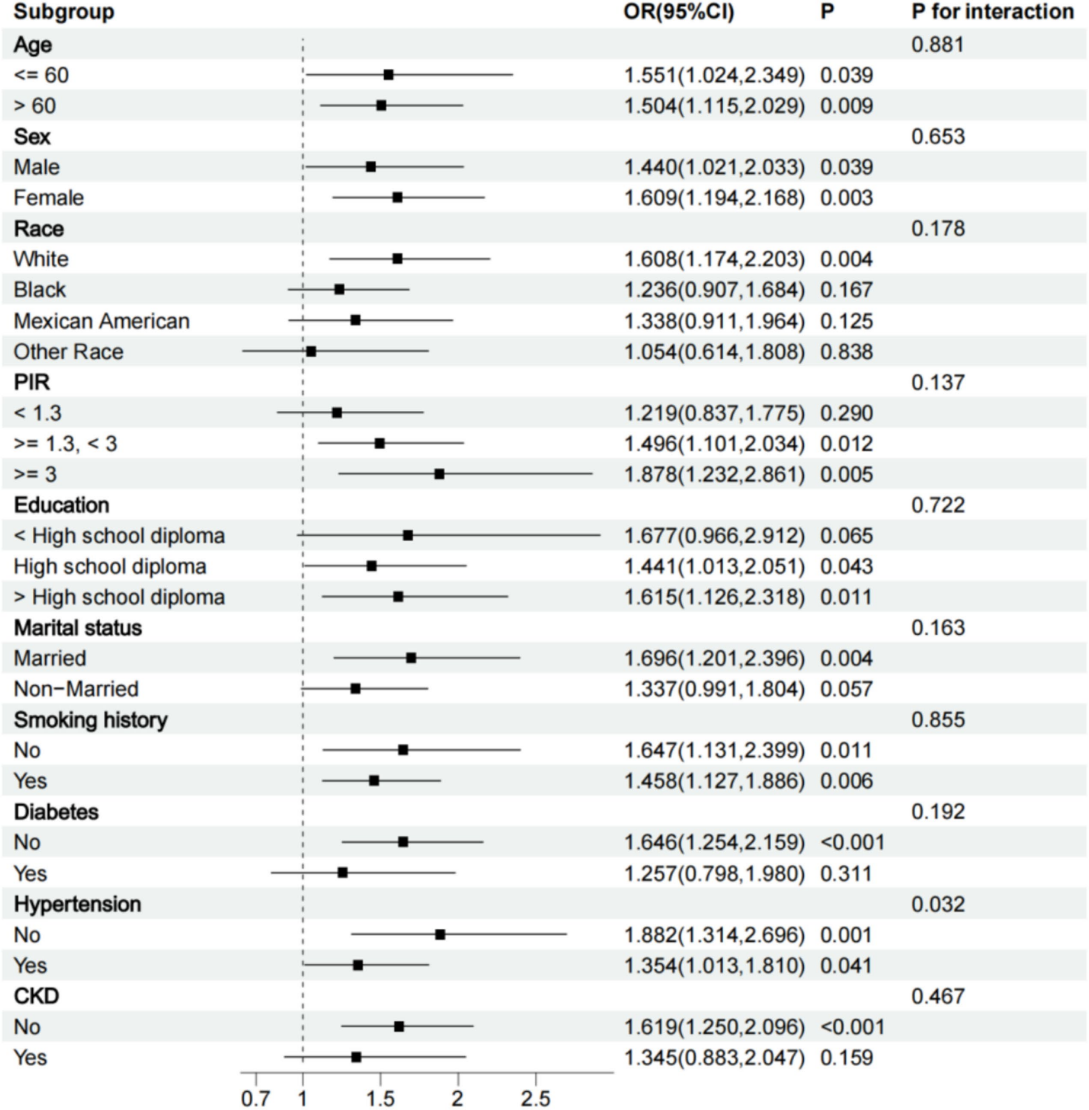
Subgroup analysis chart.

We conducted a subgroup analysis based on all grouping variables. All subgroup analyses were adjusted for all variables except the grouping variable. The results showed that the relationship between WWI and PAD remained significant in subgroups of participants older or younger than 60 years, men or women, white, with a PIR of 1.3 and above, high school diploma or above, married, those with or without a smoking history, without CKD, without diabetes, and with or without hypertension. A positive correlation trend between WWI and PAD was also observed in other subgroups. Interaction tests revealed that the positive association between WWI and the risk of PAD was particularly significant in non-hypertensive participants (P for interaction <0.05) (Figure 4).

## Discussion

In this study, we explored the relationship between WWI and the risk of PAD using data from NHANES 1999 to 2004. Through multivariate logistic regression and RCS, we found a significant positive correlation between WWI and the risk of PAD. As WWI increased, the risk of PAD also significantly rose. The ROC results showed that compared to BMI, WWI exhibited higher diagnostic efficiency and specificity in predicting the risk of PAD. The subgroup analysis results showed that the relationship between WWI and PAD remained significant in subgroups of participants older or younger than 60 years, men or women, white, with a PIR of 1.3 and above, high school diploma or above, married, those with or without a smoking history, without CKD, without diabetes, and with or without hypertension. A positive correlation trend between WWI and PAD was also observed in other subgroups.

To our knowledge, this is the first study to investigate the relationship between WWI and PAD. Previous studies on the relationship between obesity and PAD have mainly focused on using BMI as a marker of obesity (31–34). In a large cross-sectional study of the U.S. population, Sean et al. found a significant positive correlation between BMI and PAD risk, but only in women (31). Li et al., in a cross-sectional study of a hypertensive population in China, reported a U-shaped non-linear relationship between BMI and the risk of PAD, where both underweight and obese individuals had a significantly higher risk of PAD (32). Additionally, two meta-analyses showed that obese PAD patients had significantly lower long-term mortality compared to other PAD patients, which is referred to as the “obesity paradox.” (33, 34) Using traditional BMI as an indicator of obesity may overlook individuals with abdominal obesity who are at higher risk for cardiovascular diseases, which could be one of the reasons behind the obesity paradox. Compared to BMI, WWI better reflects abdominal obesity, which is a critical risk factor for metabolic and cardiovascular diseases. Qin et al. found a significant positive correlation between WWI and abdominal aortic calcification scores (35). Fang et al. reported significant positive correlations between WWI and the risks of congestive heart failure, angina, coronary heart disease, and stroke (25). Sepehr et al. found a significant positive correlation between WWI and the risk of cardiovascular disease in the general population of Iran, and WWI has a better predictive ability for cardiovascular disease than BMI (36). Our study contributes additional evidence to support the use of WWI as a predictor of the risk of cardiovascular disease.

The potential mechanism underlying the significant positive correlation between WWI and PAD risk may be related to the effects of abdominal obesity on atherosclerosis. Abdominal obesity is closely associated with insulin resistance, inflammatory responses, and endothelial dysfunction (29, 37). These factors promote the development of atherosclerosis, which is the primary pathological basis of PAD. First, visceral fat is metabolically active and secretes large amounts of inflammatory factors such as TNF-*α* and IL-6, which induce chronic inflammation and accelerate the progression of atherosclerosis (29). Second, abdominal obesity is closely related to insulin resistance. Studies have shown that free fatty acids released from visceral fat interfere with insulin signaling, leading to insulin resistance (38). Insulin resistance not only leads to abnormal blood glucose levels but also disrupts blood lipid levels, promoting lipid deposition in the blood, which further damages endothelial function and accelerates atherosclerosis (39). Additionally, visceral fat is associated with reduced secretion of adiponectin (40). Adiponectin has anti-inflammatory and anti-atherosclerotic properties, and its decreased levels may also accelerate the occurrence of PAD (41).

The findings of this study carry significant clinical implications. First, WWI is a simple and easy-to-use indicator. It can help clinicians more effectively identify high-risk individuals for PAD, especially those with normal weight but potential abdominal obesity. Early identification allows for intervention to reduce the occurrence of severe adverse outcomes. The results also offer a new perspective for future research exploring the mechanisms linking obesity and atherosclerosis. It is undeniable that this study also has some limitations. First, given that this is an observational study, we cannot pinpoint the exact mechanism by which an increased WWI contributes to the risk of PAD. Second, our study population was limited to the general U.S. population aged 40 and above, meaning the findings may not be generalizable to other populations.

## Conclusion

This study demonstrates a significant positive correlation between WWI and the risk of PAD. This finding offers a novel perspective for clinical practice, indicating that greater attention should be devoted to assessing patients’ abdominal obesity when evaluating the risk of PAD. Furthermore, for individuals with elevated WWI, proactive measures should be implemented to reduce WWI in order to mitigate the risk of PAD.

## Data Availability

Publicly available datasets were analyzed in this study. This data can be found here: all data are publicly available at https://www.cdc.gov/nchs/nhanes/.
